# On Calomel and Opium in Rheumatism

**Published:** 1811-09

**Authors:** D. H. Davies

**Affiliations:** 27, Carburton Street, Fitzroy Square


*To the Editors of the Medical and Physical Journal.*

GENTLEMEN,

IF the following case and observation meet with your approbation, you will much oblige the writer by inserting them in your widely circulated Journal.

Mrs. W. Devonshire-street, Portland-place, aged 40, was attacked, about the commencement of April last, with acute rheumatism, in consequence of taking cold from long exposure to a copious rain. She was of a corpulent make, and had never suffered any previous attack of the disease. Prior to my being requested to visit her, she had been attended by a medical gentleman for about a fortnight, but had experienced no relief, in fact, the disease was gaining ground.

From what I could learn when I first saw her, she had been taking stimulants of the usual kind, but without benefit. She complained of wandering gnawing pains, with swelling and redness about the joints of the fingers, but particularly in the *left knee;* the swelling there was to a great extent, with a *diffused redness*; incapability of moving *that joint* in the least degree; even the motion of moving the other limb, or the shake occasioned by opening or shutting the chamber door, door, would cause *violent pain.* The distress occasioned by the pain always being *worse at night*, so that the patient received little or no rest. As I stated before, she was of a plethoric habit of body, the skin was very hot and dry, the tongue furred, and every symptom of fever. Had I seen her at the commencement of the disease I should have bled her; this, however, had been omitted. I therefore desired her to relinquish taking any more of her former medicine.

I had before observed in five cases, the *marked good effects* of the combination of calomel and opium in the acute rheumatism, as well as in inflammation of the liver, and I ordered Mrs. W. to take calomel ppt. gr. ij. opii pulv. gr. j. every four hours in jelly, followed by a saline draught; this was on the third of April. On the sixth day I saw her again, she had taken her medicine regularly, and had passed a more comfortable night than for some time past; she felt considerable less pain, and had much less heat on the skin: her pulse was reduced, and her bowels gently opened by the medicine, and an almost constant diaphoresis was kept up; I ordered this medicine to be continued every four hours, as before. On the 25th I saw her again, she had occasionally slight returns of pain, but with no violence, and for the most part she was easy; the affected knee was lessened in size, and the redness had disappeared. It would now admit of some very trifling motion. I ordered a stimulant embrocation to rub the knee with three or four times a day, and desired her to continue the medicine with calomel and opium in the same doses as before. I visited her the next day, when I found her in no pain, every symptom of fever gone, the knee less swelled and allowing of considerable more motion, the bowels regularly open, but the mouth slightly affected by the mercury. I now thought proper to discontinue the medicine she had been taking, and to substitute a tonic: I directed her to take the decoction of bark with a small dose of opium three times a day. She continued this plan for a week, at the end of which she was sufficiently recovered to undertake a journey to Edinburgh. I have since heard she has had no return of the disease.

To the above case, as well as to others I have witnessed attended with a similar result, I would wish to call the attention, and at the same time to solicit the opinion of medical practitioners on the efficacy of the combination of calomel and opium. It is not with the vain idea of arrogating any novelty to myself, that I would solicit the attention of medical men to the consideration of the subject; the observation has been made by others, but I firmly believe, from my own experience, that calomel and opium given separately will not act act in the same way as when combined. Why should not the combination of these articles produce a marked and particular effect on the constitution as well as the mixture of ipecacuanha and opium? It is well known these two articles given alone will not act in the same manner as in a state of union. To ascertain, exactly, the way in which any medicines act on the constitution, I am afraid, ever will remain a desideratum; but at any rate we are able to ascertain, by the test of experience and experiment, what effect they produce. And if we find that the union of calomel and opium possesses a decided superiority over the exhibition of those articles given separately, it is sufficient for us to know the fact without inquiring how they act upon the constitution, even if the idea I entertain, that there is a specific effect produced by *this* combination, is fallacious, yet there are many advantages derived from the exhibition of this medicine in the disease under consideration. By the calomel you induce a fresh action in the system, which ought to be given till the mouth becomes slightly sore, by this means you are able to suspend the morbid action previously existing; a gentle stimulus is kept up upon the bowels, and by the opium you relieve violent pain, and at the same time prevent the calomel acting as a purgative; thus these double purposes are answered by the exhibition of this medicine, supposing that no specific effect is produced by their union, which, however, I strongly believe to be the case, but which opinion I beg leave to submit, with deference, to the judgment of your intelligent readers.

I remain, Gentlemen,

Your obliged,

D. H. DAVIES.

*27, Carburton Street, Fitzroy Square.*

